# Community-associated Methicillin-resistant *Staphylococcus aureus* in Hospital Nursery and Maternity Units

**DOI:** 10.3201/eid1106.040885

**Published:** 2005-06

**Authors:** Simona Bratu, Antonella Eramo, Robert Kopec, Elizabeth Coughlin, Monica Ghitan, Robert Yost, Edward K. Chapnick, David Landman, John Quale

**Affiliations:** * State University of New York-Downstate, Brooklyn, New York, USA;; † Long Island College Hospital, Brooklyn, New York, USA;; ‡ Maimonides Medical Center, Brooklyn, New York, USA

**Keywords:** infection control, methicillin resistance, staphylococcus aureus

## Abstract

Community-associated methicillin-resistant *Staphylococcus aureus* (CA-MRSA) has rarely been reported in the hospital setting. We report an outbreak of 7 cases of skin and soft tissue infections due to a strain of CA-MRSA. All patients were admitted to the labor and delivery, nursery, or maternity units during a 3-week period. Genetic fingerprinting showed that the outbreak strain was closely related to the USA 400 strain that includes the midwestern strain MW2. All isolates contained the staphylococcal chromosome cassette *mec* type IV. Genes for Panton-Valentine leukocidin and staphylococcal enterotoxin K were detected in all isolates, and most contained other enterotoxin genes. Testing of nearly 2,000 MRSA isolates collected during citywide surveillance studies from 1999 to 2003 showed that ≈1% were genetically related to MW2. CA-MRSA strain MW2 has been present in this region at least since 1999. This study documents the spread of this strain among healthy newborns at 1 hospital.

Methicillin-resistant *Staphylococcus aureus* (MRSA) is an established pathogen in most healthcare facilities. Recently, infections due to MRSA have been documented in children and adults who lack traditional risk factors (1–4). Most infections caused by these community-associated (CA) MRSA appear to involve the skin. However, these strains may occasionally cause pneumonia or death in previously healthy patients (5,6). In one of the initial reports of CA-MRSA, 4 deaths were reported in children infected with a prototypical strain designated MW2 (5).

Several lines of evidence suggest that the emerging CA-MRSA isolates are distinct from typical nosocomial strains (7–9). First, CA-MRSA isolates are generally susceptible to non-β-lactam antimicrobial agents and genetic fingerprinting suggests that they are unrelated to hospital-associated strains (7–9). CA-MRSA isolates possess a small (21- to 24-kb) and mobile staphylococcal chromosome cassette *mec* type IV (SCC*mec*IV)–encoding penicillin-binding protein (8). This gene cassette has been rarely found in contemporary healthcare-associated MRSA strains. Finally, most of these strains have genes that encode for multiple virulence factors, including Panton-Valentine leukocidin (PVL) and superantigens (5,10).

Strains of CA-MRSA have recently caused infections in hospitalized neonates in the nonoutbreak setting (11). They have rarely been linked to nosocomial outbreaks. One report involving postpartum women documented hospital transmission of the strain MW2 (12). We describe an outbreak in a nursery and maternity unit involving the MW2 strain of CA-MRSA. The prevalence of strains resembling MW2 in Brooklyn, New York, is also reported.

## Materials and Methods

### Outbreak Investigation at Hospital A

From October to November 2002, a cluster of skin and soft tissue infections due to MRSA involving pediatric and maternity patients occurred at a New York City hospital. The hospital has a labor and delivery unit and 2 units that house both healthy newborns and maternity patients. Healthcare workers on these units typically care for patients on all the units. After the outbreak was recognized, the following interventions were implemented: 1) nursing and medical personnel from the involved areas were informed of the outbreak and potential modes of transmission of staphylococci, 2) contact precautions were emphasized for all patients with suspected or proven skin infections, 3) alcohol-based hand sanitizers were placed in involved areas, 4) healthcare workers from involved units were screened for nasal MRSA colonization, and 5) environmental surfaces (including cord clamps, antitheft transponders, and temperature sensors of baby warmers) were tested for MRSA contamination. Healthcare workers colonized with MRSA were treated with intranasal mupirocin and furloughed until repeat cultures were negative. To identify any other potential case-patients, letters concerning the outbreak were sent to pediatricians who cared for newborns discharged from the affected units during the outbreak period. Cases were defined as MRSA infections in patients who stayed on the labor and delivery, nursery, or maternity units at any time from October 2002 to December 2002. The medical records of the patients were reviewed for information regarding prior healthcare exposures, receipt of antimicrobial agents, underlying medical conditions, treatment, and clinical outcome.

Cultures related to the outbreak were grown on tryptic soy agar plates supplemented with 3% sheep blood; colonies consistent with *S. aureus* were identified according to standard techniques. All isolates underwent susceptibility testing with the Etest method (AB Biodisk, Solna, Sweden). Ribotyping was performed with the Riboprinter Microbial Characterization System (Qualicon, Wilmington, DE, USA), as previously noted (13). In addition, isolates of MRSA collected during the outbreak were fingerprinted by pulsed-field gel electrophoresis (PFGE), as previously described (13). PFGE results were interpreted according to known criteria (14).

SCC*mec* typing was performed by using multiplex polymerase chain reaction (PCR), under conditions described by Oliveira et al. (15). Primers to detect the *mecA* gene were included as an internal positive control (15). Multilocus sequence typing (MLST) was performed on selected isolates as described by Enright et al. (16). Bidirectional DNA sequencing of 7 amplified housekeeping genes was performed with an automated fluorescent dye-terminator sequencing system (Applied Biosystems, Foster City, CA, USA). Allelic types were assigned by using the MLST database (available from www.mlst.net).

The presence of genetic sequences encoding several staphylococcal toxins was also investigated for the outbreak isolates. Based on the previously reported distribution of enterotoxins in CA-MRSA from the United States (7), the following toxins were selected for investigation: staphylococcal enterotoxin A (SEA), B (SEB), C (SEC), H (SEH), and K (SEK). In addition, strains were screened for PVL and toxic shock syndrome toxin-1 (TSST-1). Previously published primers and conditions were used to detect sequences encoding for SEA, SEB, SEC, SEH, PVL, and TSST-1 (17–19). Genes encoding for SEK were detected with the following primers: SEK forward: 5´-TGGATCAATGGAAATCACAAAA-3´ and reverse: 5´-TTTGGTAGCCCATCATCTCC-3´ (predicted product size 287 bp). The specificity of amplification was verified by bidirectional sequencing of the product.

### Surveillance Study

The identification of MW2 in the outbreak of the neonatal-maternity unit prompted a retrospective investigation to determine the regional prevalence of MRSA resembling this strain. In 1999, 2001, and 2003, surveillance studies were performed in Brooklyn, New York. Each surveillance study involved collecting all single-patient isolates of *S. aureus* from clinical microbiology laboratories during a 3-month interval. Each study included 11–15 hospitals. Susceptibility testing was performed in the central research laboratory by using the agar dilution method according to NCCLS methodology (20). All MRSA isolates were then screened for a phenotype of susceptibility to clindamycin and ciprofloxacin (typical for MW2). Isolates possessing this susceptibility pattern underwent ribotyping and SCC*mec* typing. The study was approved by the Institutional Review Board at the State University of New York (SUNY) Health Science Center and Maimonides Medical Center.

## Results

### Outbreak Investigation at Hospital A

From October 18 to November 28, 2002, a total of 8 patients with skin and soft tissue infections due to MRSA were identified. During this period, 3.5 cases of MRSA infection occurred each month in the nursery and maternity units. In contrast, no MRSA infections had been reported from the involved units in the 10 months before the outbreak. Two patients were mothers, and 6 were neonates; in no instance were both the mother and her child infected. All had been hospitalized on an involved unit at some point from October 16 to November 6, 2002. Review of medical records showed that none of the patients had prior hospital exposure, underlying chronic medical conditions, or recent antibiotic therapy.

**Figure 1:**
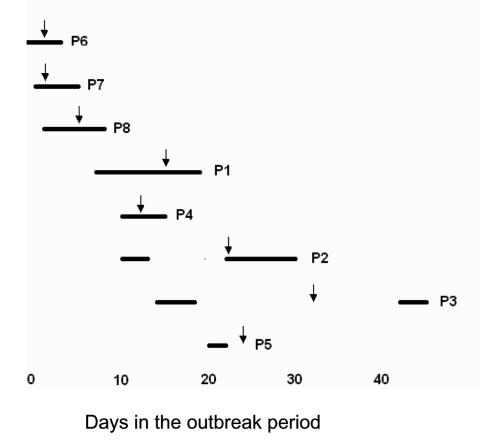
Time course of hospitalizations and onset of methicillin-resistant *Stpahylococcus aureus* illness during the outbreak at Hospital A. Solid bars represent period of hospitalization; arrows represent onset of clinical infection.

Clinical manifestations of the infections are included in Table 1. None of the patients had evidence of infection upon admission to the hospital. The timing of hospitalization and onset of clinical symptoms are shown in Figure 1. Patients stayed on the unit for an average of 5 days (range 2–12 days). Clinical infection developed in 4 of the newborns and 1 mother while in the hospital. Symptoms developed in 2 newborns and 1 mother 2, 10, and 24 days, respectively, after discharge. β-Lactam antimicrobial agents were initially administered for 6 patients. Definitive therapy generally consisted of topical or systemic antimicrobial agents active against MRSA; 1 patient required surgical drainage. All patients had clinical resolution of infection.

**Table 1:** Clinical information for patients with methicillin-resistant *Staphylococcus aureus* infection during the outbreak period

Patient	Age at onset	Sex	Strain	Infection type	Initial therapy	Definitive therapy
P1, newborn	8 d	F	USA 400	Preseptal cellulitis	Nafcillin, cefotaxime	Topical gentamicin
P2, newborn	13 d	F	USA 400	Omphalitis, otitis externa	Ampicillin, cefotaxime	Topical mupirocin
P3, mother	33 y	F	USA 400	Breast abscess	Cefazolin	Surgical drainage, vancomycin, topical mupirocin
P4, newborn	2 d	M	USA 400	Omphalitis, pustulosis	Nafcillin Gentamicin	Gentamicin, topical mupirocin
P5, newborn	4 d	M	USA 400	Pustulosis	Cephalexin	Topical bacitracin
P6, newborn	2 d	M	USA 400	Pustulosis	None	Local wound care
P7, newborn	1 d	F	USA 400	Pustulosis, mastitis	Topical mupirocin	Vancomycin
P8, mother	24 y	F	Unique	Peripheral IV catheter site	Cefazolin	Trimethoprim-sulfamethoxazole, catheter removal

Two additional suspected cases were reported by pediatricians to the Infection Control Department. The first was in an infant, born in November 2002, who was seen as an outpatient for pustulosis; however, the site was not cultured. The second case involved another infant, also born in November 2002, who was readmitted to the hospital 4 days later for treatment of omphalitis. Multiple cultures yielded no growth. No additional cases were reported from December10, 2002, to December 31, 2003.

**Figure 2:**
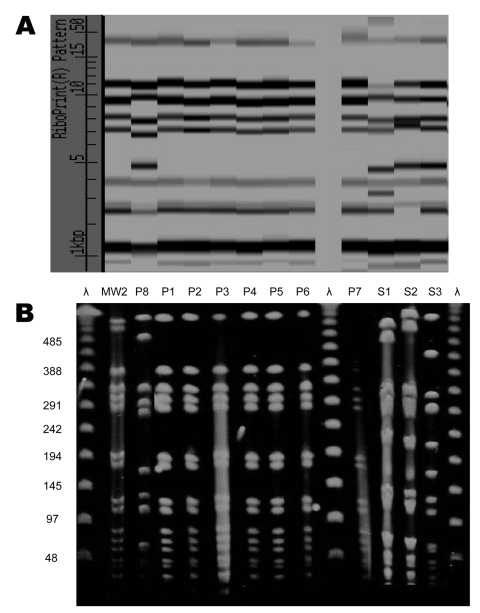
A) Ribotype and B) pulsed-field gel electrophoresis patterns of 8 clinical isolates of methicillin-resistant *Staphylococcus aureus*. Outbreak-related isolates P1–P7 are closely related to MW2. Clinical isolate P8 and the 3 isolates from healthcare workers (S1–S3) are unrelated to the outbreak strain.

Susceptibility testing showed that all 8 isolates were susceptible to clindamycin, ciprofloxacin, trimethoprim-sulfamethoxazole, rifampin, doxycycline, linezolid, and vancomycin. Of the 8 clinical isolates, 7 (isolates P1-P7) belonged to 1 ribotype that was identical to the prototypical MW2 strain (Figure 2). PFGE confirmed that the 7 isolates were identical and closely related to MW2 (Figure 2). All 7 contained SCC*mec* type IV. Since the 7 isolates appeared identical, MLST was performed on one of the isolates and showed sequence type 1. The PFGE and MLST pattern are the same as CA-MRSA clone USA 400, which also includes MW2 (21). Among these 7 isolates, all contained SEK and PVL, 6 contained SEC and SEH, and 5 contained SEA. None was found to have genes encoding SEB or TSST-1. The eighth clinical isolate, from a catheter-site infection, was distinct from the outbreak strain by ribotyping and PFGE (Figure 2). For this isolate, SCC*mec* was nontypable, and MLST typing confirmed a distinct allelic profile. None of the genes encoding toxins was detected.

A total of 189 healthcare workers worked on the involved units during the outbreak period. Screening cultures of the anterior nares were performed in 176 of the workers in November 2002. Three of the cultures were positive for MRSA, including 2 from the nursing staff and 1 from a pediatrician. The 3 MRSA strains possessed a susceptibility pattern typical for the multidrug-resistant hospital strains, with resistance to clindamycin and ciprofloxacin. They belonged to ribotypes distinct from the outbreak clone, and PFGE confirmed these isolates were unrelated to MW2 (Figure 2). For the 3 isolates, SCC*mec* was nontypable with the multiplex PCR method. None of the 27 environmental samples collected in November 2002 yielded positive cultures for MRSA.

### Surveillance Study

A total of 4,345 isolates of *S. aureus* were collected in the 3 surveillance studies conducted in 1999, 2001, and 2003; susceptibility data for these isolates are given in Table 2. A total of 1,913 (44%) isolates were methicillin-resistant. Of the 1,913 MRSA isolates, 118 (6%) possessed the screened phenotype (susceptible to both clindamycin and fluoroquinolones). Among the 118 isolates, 40 different ribotypes were identified. A total of 11 isolates possessed the same ribotype pattern as the outbreak clone, MW2. Of the 11 isolates, 4 were known to come from children. One HIV-infected adult died of overwhelming sepsis within 24 hours of hospitalization. Sources of the cultures included skin and soft-tissue in 7 patients, blood/sterile body fluid in 3 patients, and the genital tract in 1 patient. Nine of the 11 isolates had SCC*mec*IV. The number of isolates resembling MW2 remained relatively constant during the 3 surveillances (4 in 1999, 3 in 2001, and 4 in 2003).

**Table 2:** Susceptibility data on *Staphylococcus aureus* isolates collected from 11 to 15 hospitals in 1999, 2001, and 2003*

	1999 (N = 567)	2001 (N = 772)	2003 (N = 588)
% MRSA	36	46	52
Antimicrobial agent (% susceptible)			
Azithromycin	14	5	5
Clindamycin	18	15	20
Vancomycin	100	100	100
Daptomycin	ND	100	100
Tigecycline	ND	ND	100
Minocycline	98	ND	98
Linezolid	100	ND	100
Rifampin	88	92	95
Imipenem	37	49	56
Ciprofloxacin	10	8	7
Trimethoprim-sulfamethoxazole	75	80	89

*MRSA, methicillin-resistant *Staphylococcus aureus*; ND, not done.

## Discussion

This report characterizes the nosocomial transmission of the CA-MRSA strain MW2 among healthy newborns and, possibly, a postpartum woman. Symptoms developed in 3 patients 2–24 days after hospitalization; 2 may have acquired the bacteria in the hospital or the community. An eighth patient, a mother with catheter-site infection, had an unrelated strain with a pattern suggestive of a hospital-associated strain. The source of the outbreak and mechanism of transmission were not evident, as no cultures of staff members or the environment yielded this particular strain of MRSA. Transmission may have occurred after MW2 was introduced into the hospital by transient colonization of healthcare workers or by contamination of shared medical equipment. The infection control measures enacted in response to the initial cases may have had a role in controlling the outbreak. Widespread screening of healthcare workers for MRSA did not detect the outbreak strain in this and another report (12). While a potential role for this practice cannot be excluded, current evidence does not support routinely implementing widespread screening for CA-MRSA.

In the pediatric population, risk factors associated with MRSA infections include premature birth or low birth weight, chronic underlying diseases, prolonged hospitalization, invasive or surgical procedures, indwelling catheters, and prolonged use of antimicrobial agents (22–25). Outbreaks of *S. aureus* have been especially challenging in neonatal nursery units. Prior outbreaks involving the pandemic strain phage type 80/81 were characterized by high colonization rates among infants discharged from nurseries and subsequent transmission to family members (26). In this report, infection developed in the outpatient setting for 2 patients (following an admission on the involved unit), which suggests carriage of MW2 from the hospital back into the community. Unrecognized CA-MRSA colonization during hospitalization could become an additional method of its dissemination in the community.

Increased prevalence of CA-MRSA has been reported in Chicago, Los Angeles, Texas, and Minnesota (2,3,27,28). In New York City, CA-MRSA appears less common; 1 investigation reported MRSA carriage in 0.26% of children and their guardians (29). In our present report, a retrospective analysis of isolates collected from citywide surveillance studies conducted from 1999 to 2003 suggests that ≈1% of all MRSA isolates in Brooklyn are genotypically related to the prototypical North American CA-MRSA, MW2. Since only MRSA isolates that were susceptible to both clindamycin and ciprofloxacin were analyzed, this analysis probably underestimates the true prevalence. Other strains of CA-MRSA (e.g., USA 300) and USA 400 strains that acquired resistance to these antimicrobial agents would have been missed by our screening methods.

The introduction of CA-MRSA strains into neonatal units represents an especially serious challenge. Many of the infections caused by these strains, including some in our report, can be unusually severe and life-threatening (11). Careful vigilance involving surveillance, identification of these dangerous strains, and implementation of infection control measures, should be helpful in preventing further transmission both within and outside of the hospital.
